# Automated approach for quality assessment of RDF resources

**DOI:** 10.1186/s12911-023-02182-8

**Published:** 2023-05-10

**Authors:** Shuxin Zhang, Nirupama Benis, Ronald Cornet

**Affiliations:** 1 Department of Medical Informatics, Amsterdam UMC location University of Amsterdam, Meibergdreef 9, Amsterdam, The Netherlands; 2 Amsterdam Public Health, Methodology & Digital Health, Amsterdam, The Netherlands

**Keywords:** RDF, Ontologies, Linked data, Automated assessment, Data quality, URI

## Abstract

**Introduction:**

The Semantic Web community provides a common Resource Description Framework (RDF) that allows representation of resources such that they can be linked. To maximize the potential of linked data - machine-actionable interlinked resources on the Web - a certain level of quality of RDF resources should be established, particularly in the biomedical domain in which concepts are complex and high-quality biomedical ontologies are in high demand. However, it is unclear which quality metrics for RDF resources exist that can be automated, which is required given the multitude of RDF resources. Therefore, we aim to determine these metrics and demonstrate an automated approach to assess such metrics of RDF resources.

**Methods:**

An initial set of metrics are identified through literature, standards, and existing tooling. Of these, metrics are selected that fulfil these criteria: (1) objective; (2) automatable; and (3) foundational. Selected metrics are represented in RDF and semantically aligned to existing standards. These metrics are then implemented in an open-source tool. To demonstrate the tool, eight commonly used RDF resources were assessed, including data models in the healthcare domain (HL7 RIM, HL7 FHIR, CDISC CDASH), ontologies (DCT, SIO, FOAF, ORDO), and a metadata profile (GRDDL).

**Results:**

Six objective metrics are identified in 3 categories: *Resolvability* (1), *Parsability* (1), and *Consistency* (4), and represented in RDF. The tool demonstrates that these metrics can be automated, and application in the healthcare domain shows non-resolvable URIs (ranging from 0.3% to 97%) among all eight resources and undefined URIs in HL7 RIM, and FHIR. In the tested resources no errors were found for *parsability* and the other three *consistency* metrics for correct usage of classes and properties.

**Conclusion:**

We extracted six objective and automatable metrics from literature, as the foundational quality requirements of RDF resources to maximize the potential of linked data. Automated tooling to assess resources has shown to be effective to identify quality issues that must be avoided. This approach can be expanded to incorporate more automatable metrics so as to reflect additional quality dimensions with the assessment tool implementing more metrics.

**Supplementary Information:**

The online version contains supplementary material available at 10.1186/s12911-023-02182-8.

## Introduction

The Semantic Web design goal “Anyone Can Make Statements About Any Resource” [1] has resulted in an abundance of resources in RDF (Resource Description Framework) [2]. The exact size of such resources is unknown, but according to the data from the Linked Open Data Cloud website, the number of publicly available RDF resources has experienced significant growth from 12 in 2007 to 1,301 with 16,283 links in 2020 [3], which does not account for all RDF resources on the Web [4]. An RDF resource can be any structured information or knowledge in RDF, including 1) datasets and metadatasets presenting actual data and metadata, 2) schemas describing the structure of data models, and 3) ontologies formalizing concepts and relations within a specific domain. Everything other than literals [5] in an RDF resource is represented as Unique Resource Identifiers (URIs).

Among the RDF resources within the same domain, there is a large variation in the vocabularies used and the underlying data models applied, which can hamper interoperability. But such variation can be addressed through post alignment - terminology mapping [6] or semantic transformation between different data models [7]. Take for example, a data custodian having to integrate RDF resources using the Human Phenotype Ontology (HPO) with those using SNOMED CT. Ontology matching between these terminologies is needed and has been investigated [8]. Additionally, there is a large variation in the quality of RDF resources, which cannot be as easily addressed and can hinder the interoperability of resources. For example, a health data steward considers to use an RDF dataset but he finds that there is one predicate http://www.orpha.net/ORDO/Orphanet_317343 from Orphanet Rare Disease Ontology (ORDO) [9] that is non-resolvable. Consequently, neither the type of this ORDO term (such as whether it is a property) nor its semantics are known, which could lead to inconsistency and misinterpretation if the steward decides to use the dataset. Consider another example, the term http://xmlns.com/foaf/0.1/depicted_by from FOAF [10] is resolvable, but through this URI no content defining this term is found. These two examples illustrate the negative impact of quality issues on the potential of linked resources. Before employing RDF resources, it is essential to assess their quality to uncover any issues. Even more challenging is the fact that numerous factors may lead to those errors. For example, both erroneous identifiers (typos) and obsolete identifiers contribute to non-resolvability.

However, assessing the quality of linked RDF resources [11] is challenging. One major reason is that the quality of RDF resources is a multi-dimensional aspect [12–14], making it impossible for a single data custodian to identify and solve all quality issues all at once. Many researchers [15–18] have recognized this and have proposed various approaches towards quality assessment specific for linked resources. In 2011, Fürber et al. [19] pointed out the situation of missing techniques or methodologies for quality assessment relevant to the evolving “Web of Data”. To address that, they developed a rule-based framework called SWIQA, which measured five “Information Quality dimensions” that were defined by Mouzhi et al. [20]: Accuracy, Completeness, Timeliness, Uniqueness, and Semantic Accuracy. In 2012, Pablo et al. [15] developed a module called Sieve which was employed by the Linked Data Integration Framework (LDIF) [21]. They developed new metrics to measure dimensions that were defined by Bizer et al. [14]. Three metrics were demonstrated: whether a dataset contains all of the attributes needed for a given task for the Completeness dimension; whether any redundant attributes exist for Conciseness; whether any properties with cardinality 1 contain more than one value for Consistency. In 2014, Dimitris et al. [22] present a methodology for test-driven quality assessment of Linked Data, called RDFUnit. RDFUnit relies on users to define SPARQL patterns that represent constraints for certain quality dimensions, and then execute customized SPARQL queries against dataset endpoints to assess a dataset. They defined 17 patterns for checking constraints, including cardinality, whether the subject and object of a property are conformant to its “rdfs:domain” and “rdfs:range”, and whether a literal is within a given range according to users’ requirements.

Having observed various approaches for assessing linked data, Zaveri et al. performed comprehensive research on this topic in a systematic review [21, 22] in 2014. They defined 23 quality dimensions and listed one or more metrics sourced from different studies for each dimension. This comprehensive work of Zaveri et al. provides a profound theoretical foundation for future research about linked data quality assessment. In 2018, Michael et al [18] defined 34 data quality criteria and applied them to analyze and compare five knowledge graphs (DBpedia, Freebase, OpenCyc, Wikidata, and YAGO). However, they did not specify which tooling was used to conduct assessment work. Based on Zaveri’s work, Jeremy et al [23] developed a generic framework called Luzzu with the goal of being scalable, extensible, interoperable, and customizable. This framework utilizes a semantic knowledge layer, the Dataset Quality Ontology (daQ) [24], to capture the quality assessment results. Luzzu, by default, covers 14 out of Zaveri’s 23 quality dimensions and is able to implement around 60% of Zaveri’s metrics.

Except for Zaveri’s review that summarized dimensions and metrics, none of the aforementioned studies justified the selection of metrics in their approaches. For example, RDFUnit enables users to choose metrics from a predetermined metric pool, but the selection of metrics comprising this pool is not justified. This fact makes it difficult to choose appropriate metrics, because they have different characteristics. Metrics may be subjective and hence depend on human judgement (e.g., reputation); metrics may be objective but context-dependent (e.g., completeness); metrics may be easy to implement (e.g., time of last modification); or more time-consuming to assess (e.g., accurate annotation). Therefore, it is important to stratify existing metrics to support the selection of metrics. An initial selection would be a prioritized set of metrics that are straightforward, simple to apply, yet generic and essential.

In practice, numerous quality issues are encountered pertaining to the characteristics that reflect the nature of linked data: machine-actionable interlinked data on the Web. Such foundational characteristics may include syntactic correctness for a serialization format so as to enable automatic processing by machines, or explicitly relate to URIs so as to enable successful interlinking. If a resource does not possess these characteristics, the basic benefits of linked data cannot be achieved. In light of the expanding amount of RDF resources of unknown quality that have been published, manual quality assessment is not feasible. For example, the BioPortal repository now includes 984 ontologies (with over 13,000,000 total classes in total) [25].

Ontologies are the central type of RDF resources because other types (i.e., (meta)datasets and schemas) utilize terms from ontologies to enrich their own semantics. Consequently, it is essential to assess the quality of ontologies regarding the foundational aspect outlined above, particularly in the biomedical domain, which is complex and requires the use of standardized concepts/classes. Ontologies of high quality in the foundational aspects enable the timely retrieval of knowledge through the power of linked data. Nonetheless, even widely-used ontologies listed in the OBO Foundry [26] library may have these foundational issues [27].

In this paper we aim to 1) identify the objective, automatable, foundational characteristics that RDF resources must have to maximize the potential of Linked Data, and 2) introduce an approach to assessing these characteristics in a machine-actionable manner.

## Methods

In this section, we describe the process of selecting appropriate metrics as the foundational requirements that RDF resources should meet, out of those defined in existing literature, standards, and tooling. Then we illustrate how these metrics were curated and represented in a consistent way. Lastly, we present how a proof-of-concept tool was developed to demonstrate the proposed automated approach.

### Formalization of terminology

We elaborate on the most important concepts in the context in which they will be used in the remainder of this paper.

**RDF resource** - We employ the definition of “resource” from the Data Catalog Vocabulary (DCAT) [28] to describe an “RDF resource” as: “any resource published or curated by a single agent, and available for access or download in one or more RDF serialization formats”, such as Turtle [29], RDF/XML, or N3. For example, any published ontology that has an RDF representation is regarded as an RDF resource in this paper.

**Data quality** - We employ the definition of “quality of a data product” from the ISO/IEC 25012:2008 standard [30] to describe “data quality” as: “the degree to which data satisfy the requirements defined by the product-owner organization”. As an RDF resource is data, the quality of an RDF resource is a type of data quality.

**Quality dimension** - We employ the definitions [31] of Zaveri’s dimensions and adapted them to describe a “quality dimension” as: the characteristics of RDF resources that are required for reaching certain goals in a specific quality aspect. Each quality dimension can be expressed by one or more metrics. For example, *Consistency* is a quality dimension that requires an RDF resource (including the underlying data model and used vocabularies) to be “free of contradictions”. Using deprecated classes in specifications of non-deprecated subjects is an example of a “contradiction”.

**Quality Metric** - We employ the definition of “metric” from the Data Quality Vocabulary (DQV) [32] to describe a “quality metric” as a quality indicator to reflect a quality dimension. For example, the extent to which deprecated classes are used is one of the metrics for expressing the dimension Consistency.

### Materials

We utilize existing literature, tools, and standards to draw up an initial list of metrics that reflect the data quality of RDF resources in various aspects. We derived the initial list from the following materials, which are the most representative of related work:

**Zaveri’s systematic review** - this research work defined ninety-six metrics reflecting twenty-three quality dimensions for linked data quality assessment [31]. This study is chosen because it summarized the research work relevant to linked data quality assessment from 2002 to 2012. So far it is still the most recent systematic review in this field and have been widely cited.

**ISO/IEC 25012:2008** - this standard [30] defines the Data Quality Model that describes fifteen characteristics that should be taken into account when assessing a data product. This standard is selected because it is the most relevant standard to linked data quality assessment, though these fifteen characteristics are relatively high-level compared to concrete metrics.

**Luzzu** - this quality assessment tool implements forty-four metrics, some of which are Zaveri’s metrics [31]. The metrics implemented in this tool are described in the Linked Data Quality Metric (DQM) [33] vocabulary and represented in RDF. This tool is chosen as source for the initial list of metrics because it is the newest tool developed and still maintained to perform quality assessment of linked data.

### Selection of metrics

To enable automated assessment of the foundational characteristics of an RDF resource, a set of appropriate metrics was selected according to the following criteria:

1. A metric should be **objective**, i.e., its outcome is independent of the assessor.
2. A metric should be **automatable**, i.e., the measuring process can be automated. One premise of being automatable is being generic and applicable to most real-world cases. Consider an objective metric for detecting whether trust-related feedback from users is provided. It is not automatable because no standardized property is pre-specified to declare such information, so different communities may use different properties to store this information: one uses the property ‘rdfs:comment’ from the RDF Schema (RDFS) [34] while another uses ‘dqv:UserQualityFeedback’ from DQV [32].
3. A metric should be **foundational** so that it can reflect the basic characteristics an RDF resource must have. As such, a foundational metric should focus on low-level assessment around the basics for Linked Data - URIs, semantics, and machine-actionability. If any quality issue is identified in an RDF resource while testing a foundational metric, the benefits of Linked Data to this resource is limited.

### Representation of metrics

To describe selected metrics in a consistent way, those metrics, originally from different sources, were curated, including deduplication, merging similar metrics, and refining definitions considering the RDF resource context, e.g., change from ‘dataset’ to ‘resource’.

Metrics addressing the same aspect were grouped into a quality category if necessary. A quality category is defined as a curated collection of objective quality metrics reflecting similar quality issues, with a narrower scope than a quality dimension.

Curated metrics were represented in RDF and semantically aligned to existing quality dimensions from the Linked Data Quality Dimension (LDQD) vocabulary [35] through properties from the Simple Knowledge Organization System (SKOS) [36] and DQV [32].

### Proof-of-concept

To provide practical support to the proposed approach aimed at establishing the foundational quality requirements for (reuse of) RDF resources, we developed a tool as a proof-of-concept to assess resource quality through testing selected metrics. The tool was developed in Python utilizing the rdflib package [37], and is available on Github [38]. The pseudocode for this tool is described in the Additional file 3.

We performed the quality assessment on eight RDF resources from generic to specific on July 5th 2022:

- **Metadata Profile of Gleaning Resource Descriptions from Dialects of Languages (GRDDL)** [39]. GRDDL is a technique enabling users to obtain RDF triples out of XML documents, so predicates from its metadata profile are used by other RDF resources, for example OWL.
- **Dublin Core**^**TM**^ **Metadata Initiative Metadata Terms** (DCT) are the fifteen terms of the Dublin Core^TM^ Metadata Element Set (also known as ‘the Dublin Core’) plus several dozen properties, classes, datatypes, and vocabulary encoding schemes. These metadata terms are expressed in RDF vocabularies for use in Linked Data [40].
- **Friend of a Friend(FOAF)** [10] is a commonly-used ontology which ‘describes persons, their activities and their relations to other people and objects’ (quoted from https://en.wikipedia.org/wiki/FOAF_(ontology). Many Semantic Web specifications and guidelines have utilized this ontology to create examples, such as https://www.w3.org/TR/turtle/ and http://shex.io/shex-primer/.
- **Semanticscience Integrated Ontology (SIO)** [41] is ‘a simple, integrated ontology of types and relations for rich description of objects, processes and their attributes’. Its RDF representation and more detail can be found at https://github.com/MaastrichtU-IDS/semanticscience.
- **HL7 Reference Information Model (RIM)** is a static conceptual model developed by Health Level 7 (HL7) [42] for healthcare data. Although this model is based on the Unified Modelling Language (UML), an RDF rendering is available.
- **Fast Healthcare Interoperability Resources (FHIR)** [43] is a standard developed by HL7 for data exchange in healthcare. While generally provided in JSON or XML, an RDF rendering is available as well.
- **The Clinical Data Acquisition Standards Harmonization (CDASH)** [44] defines ‘a standard way to collect data across studies and sponsors, so that data collection formats and structures provide clear traceability of submission data into the Study Data Tabulation Model (SDTM) in the Clinical Data Interchange Standards Consortium’.
- **The Orphanet Rare Disease ontology (ORDO)** [9] is a structured vocabulary specific for rare diseases. It is recommended by the European Commission Joint Research Center to describe diseases, genes and other relevant features in the rare disease field.

Any quality issue identified during quality assessment was regarded as an error. For quantitative analysis, the number of errors and the proportion of erroneous triples were calculated. These results can serve as evidence that selected metrics can be operationalized.

## Results

In this section, we present the results of the metric selection process, and the operationalization of the selected metrics in RDF. We also present results from the assessments we made on the selected resources.

### Metric selection process

**Fig. 1:**
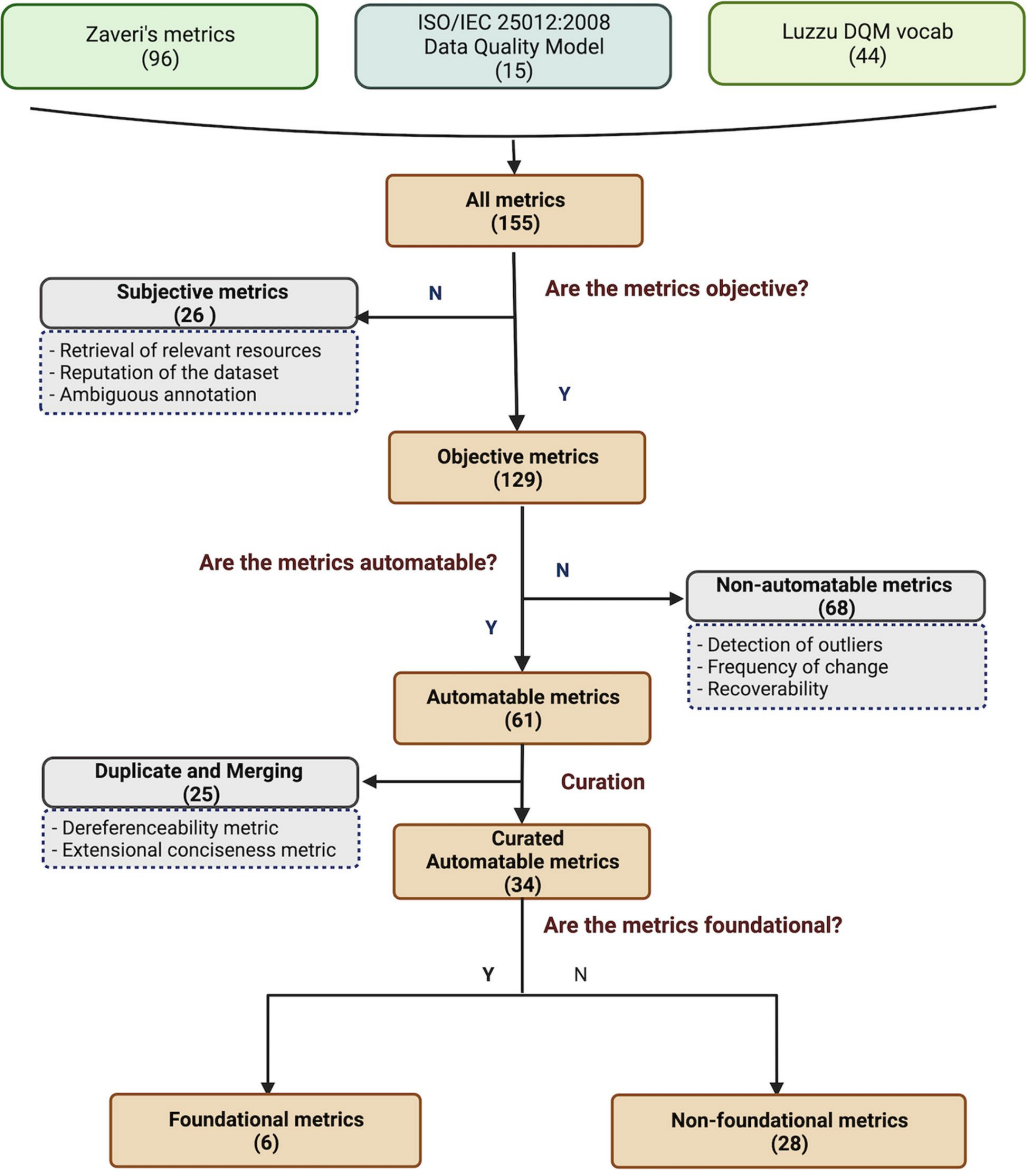
Selection of quality metrics. Examples of excluded metrics are shown in the dashed boxes. More detail can be found at Additional file 1

There are 155 metrics drawn from Zaveri’s systematic review (96), Data Quality Model of ISO/IEC 25012 (15), and the DQM vocabulary (44), reflecting thirty-seven quality dimensions, such as *Availability, Consistency, Licensing, and Reputation*, see Fig. 1. More details can be found in the Additional file 1.

The majority of the metrics are objective (129), while the remainder (26) are subjective. ‘Detection of ambiguous annotation’ and ‘reputation of an RDF resource’ are examples of subjective metrics that require the interpretation and input of users. Less than half of the objective metrics (61 out of 129) are automatable and allow for automated assessment. An example of an automatable metric is to calculate the number of answered HTTP-requests per second to reflect high throughput performance for an RDF resource, which is a special metric in the context of Linked Data that is system-dependent [30].

These 61 automatable metrics include 31 Zaveri’s metrics in 15 quality dimensions, 24 Luzzu DQM metrics in 10 dimensions, and 6 ISO/IEC 25012:2008 metrics in 6 quality dimensions (see Additional file 1). After curation, twenty-five metrics were removed, including twenty-three duplicates and two merged metrics. These duplicate metrics have the same meaning, but they are expressed differently in various sources. To ensure that as many metrics as possible are expressed in the same manner (e.g., the writing style), duplicate metrics from DQM and ISO/IEC 25012:2008 were eliminated, and only metrics from Zaveri were retained, which comprise the majority of metrics. The metrics ‘accessibility of the RDF dumps’ and ‘dereferencability issues’ were merged and curated as ‘non-resolvable URIs’. The metrics ‘application of content negotiation’ and ‘no structured data available’ were merged and curated as ‘non-parsable URIs’. The metric ‘application of content negotiation’ examines whether data can be retrieved in accepted formats via a URI, including free texts, whereas the metric ‘no structured data available’ examines whether structured data (particularly in RDF) is available, excluding free texts and omitting technical aspects. Both metrics require content negotiation on the RDF serialization formats but are described at distinct levels. Therefore, they are not considered duplicates but rather address similar issues. These two merged metrics, the ‘Basic Provenance Metric’ from DQM, and three metrics ‘Compliance’, ‘Efficiency’, and ‘Portability’, altogether six metrics, are not included in Zaveri’s list of quality metrics.

The remaining 34 (see Additional file 2) curated automatable metrics, reflecting eighteen quality dimensions, can serve as the cornerstone of the automated approach in this paper on RDF resources, though the current focus is on the foundational qualities. Out of these automatable metrics, only six are foundational. Their definitions are provided in the next subsection.

### Quality metrics reflecting foundational requirements

**Fig. 2:**
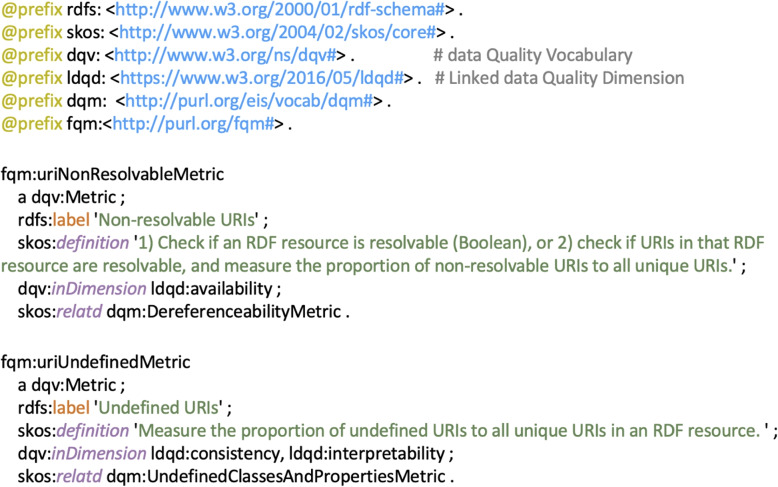
An excerpt of the Foundational Quality Metrics (FQM)

Six selected metrics, selected according to the criteria: objective, automatable, and foundational, were organized into three categories, *Resolvability, Parsability and Consistency*, see Table 1. An excerpt of their Turtle representation is shown in Fig. 2. Complete RDF rendering can be found at https://purl.org/fqm#. Each metric is illustrated with a specific example, see Table 2.

**Table 1:** List of metrics as minimal requirements for quality assessment on RDF resources

Category	Metric	Definition
Resolvability	Non-resolvable URIs	Measure the proportion of unique non-resolvable URIs to all unique URIs in an RDF resource. A URI is non-resolvable if it returns an error code (e.g., http 404).
Parsability	Non-parsable URIs	Measure the proportion of unique non-parsable URIs to all unique URIs in an RDF resource. A URIs is non-parsable if its media type is indicated as RDF content-type, but its content cannot be parsed as RDF triples.
Consistency	Undefined URIs	Measure the proportion of unique, undefined URIs to all unique URIs in an RDF resource. A URI is considered as undefined if it does not exist within the parsed RDF triples resulting from resolving the URI.
	Misplaced classes or properties	1)Measure the proportion of classes which are incorrectly used as a predicate to all unique classes; or 2) measure the proportion of properties which are incorrectly used as a class to all unique properties.
	Misuse of owl:DatatypeProperty or owl:ObjectProperty	Measure the proportion of misused ‘owl:DatatypeProperty’ (or ‘owl:ObjectProperty’ ) properties to all properties.
	Use of deprecated classes or properties	Measure the proportion of deprecated classes or properties to all unique classes or properties.

**Table 2:** Example quality issues to be identified by metrics. * denotes that these are parts of the metrics divided to provide clear examples. For example, the metric “misplaced classes and properties” is divided into “misplaced classes” and “misplaced properties”. The examples without ‘Resource’ are synthetic and used for demonstrating the usage of metrics

(Part of) Metric	Example	Explanation
Non-resolvable URIs	Resource: http://semanticscience.org/ontology/sio.owl Example triple: http://semanticscience.org/resource/SIO_001238http://semanticscience.org/resource/similarTohttp://purl.obolibrary.org/obo/OBI_0000052.	The URI http://semanticscience.org/resource/similarTo is expected to provide more information about the property but returns “404 NOT Found”. So the URI is non-resolvable.
Non-parsable URIs	Resource: https://raw.githubusercontent.com/ejp-rd-vp/resource-metadata-schema-ontology/main/ejprd_resource_metadata_ontology.owl Example triple: http://www.andrea-perego.name/foaf/#mehttp://www.w3.org/1999/02/22-rdf-syntax-ns#typehttp://www.w3.org/2002/07/owl#NamedIndividual.	The subject URI http://www.andrea-perego.name/foaf/#me returns structured text with the content-type “application/rdf+xml”, which is one of the content-types that supports RDF format. However, this URI cannot be parsed to RDF triples, and therefore is categorized as non-parsable. In this case, the failure is attributed to the misuse of “rdf:ID” as invalid non-colonized name (NCName) to cause a parse error.
Undefined URIs	Resource: https://obofoundry.org/registry/ontologies.ttl Example triple: http://purl.obolibrary.org/obo/bfohttp://purl.org/dc/terms/1.1/theme ’upper’.	The property http://purl.org/dc/terms/1.1/theme is resolvable and parsable, returning RDF triples of the entire DCT ontology. However, the property itself does not exist within the parsed RDF and hence is undefined.
Misplaced classes*	Resource: https://ceur-ws.org/Vol-1312/ldop2014_paper3.pdf Example triple: http://m-culture.in.th/48081http://purl.org/dc/dcmitype/Imagehttp://m-culture.in.th/media/big/%20201945.jpeg.	The URI, http://purl.org/dc/dcmitype/Image, is defined as a class, so it should not be used in the example triple as a predicate and is recognized as a misplaced class.
Misplaced properties*	Example triple: https://creativecommons.org/licenses/by/4.0/http://www.w3.org/1999/02/22-rdf-syntax-ns#typehttp://purl.org/dc/terms/license.	The URI, http://purl.org/dc/terms/license, is defined as an “rdfs:Property”. So it should not be used as the object of an “rdf:type” triple, and recognized as a misplaced property.
Misuse of owl:ObjectProperty*	Resource: http://purl.obolibrary.org/obo/cido.owl Example triple: http://purl.obolibrary.org/obo/OAE_0001045http://www.w3.org/2000/01/rdf-schema#seeAlso “NCIt:C78345”.	As an owl:ObjectProperty, the object of the property http://www.w3.org/2000/01/rdf-schema#seeAlso should be a linked resource rather than the string “NCIt:C78345”. So in this example, this property is misused.
Misuse of owl:DatatypeProperty*	Resource: https://github.com/ejp-rd-vp/CDE-semantic-model/wiki/Personal-information Example triple: http://purl.org/ejp-rd/cde/v010/example-rdf/gender1http://semanticscience.org/resource/SIO_000300http://purl.bioontology.org/ontology/SNOMEDCT/703118005.	As an owl:DatatypeProperty, the object of the property http://semanticscience.org/resource/SIO_000300 should be literal (e.g., integer or string) rather than a linked resource concerning SNOMED CT. So in this example, the property is misused.
Use of deprecated classes or properties*	Example triple: http://ncicb.nci.nih.gov/xml/owl/EVS/Thesaurus.owl#C50680http://www.w3.org/2004/02/skos/core#relatedhttp://purl.obolibrary.org/obo/VSAO_0000002 .	The class, http://purl.obolibrary.org/obo/VSAO_0000002, has a deprecation-related triple: http://purl.obolibrary.org/obo/VSAO_0000002http://www.w3.org/2002/07/owl#deprecated “true”⌃⌃xsd:boolean . It indicates that this class is deprecated and any use of it is regarded as an error counted by this metric. The same applies to properties.

The metric ‘Non-resolvable URIs’ in the *Resolvability* category 1) checks if the tested RDF resource is resolvable given the URI input, and 2) checks if URIs in that RDF resource are resolvable, and measures the proportion of non-resolvable URIs to all URIs used in that RDF resource.

The metric ‘Non-parsable URIs’ in the *Parsability* category 1) checks if the RDF resource itself can be parsed into RDF triples (i.e., if parsable), and 2) checks if URIs in that RDF resource are parsable, and measures the proportion of non-parsable ones to all URIs.

To measure the ‘Undefined URIs’ metric in the *Consistency* category, for the URIs that are parsable, their retrieved RDF content is screened for the definitions of the URIs themselves. If the URIs are not defined, it is considered an error. These undefined URIs either refer to other RDF datasets (usually as subjects in a triple) or ontological terms. The latter case is useful to identify the poorly-defined concepts in an ontology and improve the quality of the ontology accordingly.

While the previously mentioned metrics check all URIs within an RDF resource, the remaining three metrics in the Consistency category examine the correct usage of terms, i.e., classes and properties, from ontologies. The metric ‘Misplaced classes or properties’ checks if a class is incorrectly used as a predicate or if a property is incorrectly used as the object of an rdf:type triple. The ‘owl:DatatypeProperty’ properties are defined in Web Ontology Language (OWL) [45] as the properties relating objects to datatype values (or literals [5]); the ‘owl:ObjectProperty’ properties are defined as the properties relating objects to other objects (or URIs). The metric ‘Misuse of owl:DatatypeProperty or owl:ObjectProperty’ checks if an ‘owl:DatatypeProperty’ property used as a predicate is followed by a literal and if an ‘owl:ObjectProperty’ property used as a predicate is followed by a URI, otherwise this property is misused. The metric “use of deprecated classes or properties” checks if any terms deprecated by the defining ontology are used.

### Proof-of-concept

These six selected metrics were implemented in the proof-of-concept tool and tested on the eight selected RDF resources. This tool’s quality assessment procedure for each resource consists of these steps (see pseudocode in the Additional file 3):

1. The tool extracts all unique URIs from an RDF resource, retrieves their HTTP status codes (the “non-resolvable URIs” metric), and identifies non-resolvable URIs if their status codes are “4xx client error” or “5xx server error”.
2. The tool gathers the content-type of all resolvable URIs and classifies them into URIs with or without RDF content-type. RDF content-type is the MIME media Types [46] corresponding to common serialization formats of RDF, see their mappings in Table 3. URIs with RDF content-type are evaluated, and recorded as errors if they cannot be parsed into RDF triples (the “non-parsable URIs” metric).
3. From all parsable URIs identified in step (2), the tool examines whether each URI is defined in its parsed RDF triples, i.e., being a subject at least in one of these triples. If a parsable URI is not defined there, it is deemed as an error (the “undefined URIs” metric). This step relies on pattern matching and thus could be sensitive to syntax.
4. Out of all defined URIs, the tool extracts classes (URIs of type ‘owl:Class’ or ‘rdfs:Class’ without any property type) and properties (URIs of ‘rdf:Property‘ or any OWL properties), and checks whether their deprecation are ‘true’ by the property ’owl:deprecated’.
5. The tool analyzes the usage of all non-deprecated terms (the ‘misplaced classes or properties’ metric and the ‘misuse of owl:DatatypeProperty or owl:ObjectProperty’ metric) to determine whether they are correctly used. ‘misplaced classes’ refers to the classes used as predicates in triples; ‘misplaced properties’ refers to the properties which are the objects of rdf:type triples.
6. The tool generates an assessment report in CSV format that includes erroneous URIs and their proportions to all URIs. The time cost of quality assessment is recorded for each resource.

**Table 3:** Mappings between RDF serialization formats and Common MIME types

RDF Serialization Format	RDF Content-type
Turtle	text/turtle, application/x-turtle
N-Triples	text/plain
JSON-LD	application/ld+json
Notation 3	text/n3
RDF/XML	application/rdf+xml
RDF/JSON	application/ld+json

### Assessment results

After assessing the eight open RDF resources with the developed tool on July 5th 2022, only tests on two metrics revealed quality issues: ‘non-resolvable URIs’ and ‘undefined URIs’, see Table 4.

**Table 4:** Number of errors identified by automated tool with proportions and their affected triples. Assessed on July 5th 2022. The remaining four metrics are not included as no quality issues are identified in these metrics

RDF resources	Non-resolvable URIs		Undefined URIs		Time Cost (minutes:seconds)
	# URI (%)	# Affected Triples (%)	# URI (%)	# Affected Triples (%)	
DCT	1/28 (3.6%)	1/107 (0.9%)	0	0	2:09
SIO	8/1,889 (0.4%)	94/15,608 (0.5%)	0	0	22:41
FOAF	78/113 (69%)	622/631 (98%)	0	0	1:01
HL7 RIM	118/137 (86%)	520/636 (82%)	8/137 (5.8%)	252/636 (40%)	0:53
HL7 FHIR	6,762/6,930 (97%)	53,652/67,907 (79%)	1/6,930 (0.0%)	1/67,907 (0.0%)	27:06
GRDDL	9/46 (19%)	9/74 (12%)	0	0	1:50
CDASH	33/54 (61%)	184/184 (100%)	0	0	0:24
ORDO	53/15,070 (0.3%)	162,684/1,142,401 (14%)	0	0	150:65

All resources tested have non-resolvable URIs with their proportions ranging from 0.3% (for ORDO) to 97% (for HL7 FHIR) at the time we tested these resources. These non-resolvable URIs have affected triples in each RDF resource ranging from 0.5% (in SIO) to 100% (in CDASH Metadata Model). Only HL7 RIM (8 out of 137), and HL7 FHIR (1 out of 6,754) have undefined URIs, of which examples are as shown in Table 5.

**Table 5:** Examples of undefined URIs

RDF Resource	Undefined URI	Comment
HL7 RIM	http://www.w3.org/1999/02/22-rdf-syntax-ns#domain	The term ’domain’ is defined in ‘rdfs:domain’ (https://www.w3.org/1999/02/22-rdf-syntax-ns#domain) rather than ‘rdf:domain’
	http://purl.org/dc/elements/1.1/terms	The term ‘dct:terms’ does not exist.
	http://open-services.net/ns/core#Zero-to-many	This term does not exist,probably should be http://open-services.net/ns/core#Zero-or-many.
HL7 FHIR	http://build.fhir.org/fhir.ttl	This URI appears only once as an object, which requires users to further investigate.

Although the URI http://build.fhir.org/fhir.ttl is identified as an undefined by this tool, this is not necessarily an error but require users to further investigate and make the decision.

The health data models (i.e., HL7 RIM, FHIR, and CDISC CDASH) have higher proportion of non-resolvable URIs than the assessed ontologies (i.e., DCT, SIO, ORDO). Most non-resolvable URIs in those models are the new terms in the models, for example http://hl7.org/orim/class/Entity in HL7 RIM, http://hl7.org/fhir/url in HL7 FHIR, and http://rdf.cdisc.org/mms#Dataset in CDISC CDASH. A possible exaplanation is that the RDF representation of these data models are still in the development phase on RDF representation.

The time for automated assessments ranged from 24 seconds for CDASH Metadata Model to over 150 minutes for ORDO. The assessment time seems to partially depend on the total number of resolvable or parsable URIs.

## Discussion

In this paper, we extracted six objective, automatable, and foundational metrics out of existing literature. They reflect the basic characteristics that an RDF resource should possess to utilize the potential of Linked Data. The proof-of-concept tool, based on these metrics, identified a considerable number of non-resolvable URIs in all eight open RDF resources tested, and undefined URIs in two resources.

### Strengths and limitations

We can identify several strengths and limitations of our efforts in developing this automated approach. First of all, a major strength is that our approach is established on objective metrics, which maximally enables automated assessment by machines. Another strength is that through our selection criteria (i.e., objective, automatable, and foundational), we prioritized quality metrics for RDF resources that were extracted from literature. This selection and prioritization can provide guidance for those who have difficulties in determining which metrics should be tested ahead of others. In addition, reusing existing materials for pooling metrics, representing metrics in RDF, and aligning them to existing metrics all facilitate interoperability and machine-readability.

The metric characterization and selection process were conducted by the first author and reviewed by the other authors. The small size of the review group may lead to a different ‘classification’ of a metric than other reviewers from different perspectives, so we consider this a limitation of our approach. Additionally, the implementation of metrics is dependent on practice, and in this paper, the implementation is performed by the proof-of-concept tool by means of basic algorithms. Taking the “misplaced classes or properties” metric as an example, the tool determines “misplaced properties” as the properties that are the objects of ‘rdf:type’ triples. But in reality, there are more “misplaced” scenarios. For example, if a property P is defined in the RDF resource A but is used as a subject in another RDF resource B, then this property can be regarded as ’misplaced’. This scenario is not included in the current tooling but is to be implemented.

### Related work

Table 6 provides an overview of the existing approaches to be compared with our approach. A significant difference is that our approach proposed a set of concrete criteria so as to select metrics that are objective, automatable, and foundational, while other approaches selected a mixed set of metrics. For example, SWIQA not only implemented the subjective metric “Semantic Accuracy” but also the objective metric “Timeliness”. Sieve only implements three objective metrics. “Completeness” requires extra input for “gold standard” as complete, which is often domain- or user-dependent and not automatable while the metric about checking cardinality for “Consistency” is automatable. In RDFUnit, most metrics are related to triple constraints (e.g., a resource should only have one ‘rdfs:label’) and node constraints (e.g., ‘dbo:height’ of a‘ dbo:Person’ is not within [0.4,2.5]) and require specific input from users to build constraint rules. In Luzzu, 25 metrics are implemented, including four out of the six foundational metrics we selected (except ‘non-parsable URIs’ and ‘undefined classes and properties’). However, Luzzu focused on the establishment of the semantic framework and demonstrating it through those metrics rather than tackling a specific aspect or use case of linked data quality assessment. More detail and examples can be found in Table 6.

**Table 6:** Comparison of proposed automated approach to other existing framework. The ’Novelty’ of each framework in this table are not comprehensive and only include relevant content to enable comparison

	SWIQA (2011)	Sieve (2012)	RDFUnit (2014)	Luzzu (2016)	Our approach (2022)
Purpose	Proposal of a framework for information quality assessment of Semantic Web data	Proposal of a Linked Data Quality Assessment and Data Fusion module employed by the Linked Data Integration Framework (LDIF)	Proposal of a framework based on the data quality integrity constraints (represented in SPARQL patterns)	Proposal of a semantic framework based on Dataset Quality Ontology (daQ)	Proposal of an automated approach to assess the foundational characteristics as the starting point for linked data quality assessment
Scope of metrics	Mixed (5)	Mixed (3)	Mixed (17)	Mixed (25)	Only foundational (6)
Example metric	Legal Value Range Rules	Completeness	TYPRO-DEP: A resource of a specific type should have a certain property.	Basic Provenance and Extended Provenance	Non-resolvable URIs
Example or explanation	’The property foo:population must only contain values greater than zero. ’	“In the use case described in this paper, the task required retrieving 3 attributes (areaTotal, foundingDate, populationTotal) for all 5565 objects (Brazilian municipalities).”	’a dbo:Person should have a dbo:birthDate’	’if a dataset, usually of type void:Dataset or dcat:Dataset, has the most basic provenance information; that is information related to the creator or publisher, using the dc:creator or dc:publisher properties. ’	’1) Check if an RDF resource is resolvable (Boolean), or 2) check if URIs in that RDF resource are resolvable, and measure the proportion of non-resolvable URIs to all unique URIs. ’
Link	Not found	https://github.com/wbsg/sieve	https://github.com/AKSW/RDFUnit	https://github.com/Luzzu/Framework	https://github.com/sxzhang1201/assess-rdf-resource
Tooling last update	Not found	2014	2022	2021	2022
Novelty	The first linked data quality framework	Connection with Linked Data Integration Framework (LDIF)	Use of SPARQL pattern	Add semantic layer and quality metadata to assessment framework	Focus on foundational aspects

### Implication

In real-world situations, numerous quality issues concerning foundational characteristics reflecting the nature of linked data are encountered: URIs of terms that are not machine-actionable, or inappropriately used. Meanwhile, we have found that numerous metrics are domain- or user- dependent, or require additional human input. These metrics are not useless but they may inhibit the quality evaluation process, particularly when there are large resources to be assessed. Therefore, it is necessary to conduct an automated approach to examine the foundational characteristics of RDF resources in a comprehensive way as a starting point before assessing more complex metrics.

Besides, quality assessment in the foundational aspect for RDF resources usually relates to the functioning of URIs and the direct knowledge out of them. Therefore, applying our approach can also facilitate the creation of Globally Unique, Persistent, Resolvable Identifiers (GUPRIs) [47].

Moreover, the errors identified by the tool based on our approach can provide valuable insight on factors that contribute to these errors. This can result in solutions for quality improvement and furthermore help develop guidelines for data providers. For ontologies, it is potential to incorporate such guidelines focusing on the foundational characteristics with the existing guidelines [48–50]. Two factors are identified that may contribute to the foundational quality issues:

- Confusion between similar ontologies. One undefined URI in HL7 RIM (http://www.w3.org/1999/02/22-rdf-syntax-ns#domain) might be attributed to the confusion between http://www.w3.org/1999/02/22-rdf-syntax-ns# and http://www.w3.org/2000/01/rdf-schema#. Such confusion might be avoided by using Protege [51] where ‘rdf’ and ‘rdfs’ are built-in vocabularies and external ontologies can be imported. Similar confusion may also occur between ‘dct’ and ‘dcat’. So using Protege can be an advice included in guidelines to avoid or address these errors.
- Dynamic resolvability. All the terms defined in FOAF were not resolvable at the assessment time (July 5th 2022) but before that they had been resolvable, which however is not rare in the field utilizing linked resources. Therefore, it is important to take such dynamicity into account when generating guidelines. Example considerations may include: whether to use an ontology that is frequently non-resolvable without warning; whether to drop a commonly-used ontology that is periodically non-resolvable due to maintenance.

Additionally, the representation of the foundational metrics in RDF at https://purl.org/fqm# can serve as a template for new metrics to be represented in RDF in a consistent way. For example, use the class ‘dqv:metric’ to instantiate a quality metric and the property ‘dqv:inDimension’ to refer to the corresponding quality dimension from the DQV vocabulary [32].

### Future work

Our approach provides a theoretical basis for quality assessment of RDF resources with foundational metrics. Hence, we plan to apply this approach to assess RDF resources in a specific domain, namely rare diseases, and we believe that the assessment results can be used to produce domain-specific recommendations for RDF resource providers. The automated approach will be extended with other metrics that are automatable. These foundational metrics are a start, necessary but not sufficient. Other dimensions such as Licensing are of importance and ’provision of machine-readable indication of a license’ has the potential to be automated.

While the foundational metrics used in this paper are equally weighted, other metrics to be added in the future may not all have the same importance. Metrics that are not foundational but automatable could be weighted differently depending on the context of the assessment, e.g., taking data consumers’ requirements and preferences into account. For a data aggregator (or a person working on data aggregation) who collects information for a given task, metrics in *Availability* (from Zaveri’s work) might be the most important aspect to enable compiling information from various sources. For a data modeler who works on the information mapping between two different systems, metrics in the *Interpretability* and *Conciseness* dimensions (from Zaveri’s work) might be of high importance.

As for tooling, the current version is mainly for proof-of-concept, thus, testing, evaluating, and refining this tool is needed for its generalizability. Additionally, we intend to expand our assessment tool by implementing the remaining automatable metrics as identified in the results with the potential integration with external tools specialized in certain tasks. For example, RDF doctor [52] is a tool dedicated to detecting and resolving syntactic errors in a semi-automatable fashion. Furthermore, RDF resources produced in different scenarios will be assessed, such as RDF resources generated by an RDF transformation tool: OpenRefine [53], or a commonly-used ontology generator: Protege [51]. Besides, there is room for users’ feedback regarding either the approach or the tool, which is essential not only to help establish a community for disseminating quality assessment, but also to keep in touch with the practical needs of data quality in RDF resources. To further support this, generating report in RDF to capture quality-related metadata is needed, for example by incorporating DQV vocabulary including user feedback (‘dqv:UserQualityFeedback’) and the PROV ontology [54] including assessment time (‘prov:generatedAtTime’).

## Conclusion

In this paper, we identified six objective metrics regarding *Resolvability, Parsability,* and *Consistency* as the foundational quality requirements of RDF resources to utilize the benefits of Linked Data, regardless of domain and resource type. The developed tool, which implements these metrics, as a proof-of-concept, identified non-resolvable URIs and undefined URIs in eight RDF resources. To further support the application of our automated approach, we plan to include more automatable metrics and to further develop the tooling to enable those new metrics to be implemented.

## Supplementary Information

**Additional file 1.** Metric Pool. Additional file 1 lists all 155 metricswith their attributes including index, quality dimension, metric name, definition, source, whether it is objective, whether it is automatable, whether it is foundational, and comment.

**Additional file 2.** List of automatable Metrics. Additional file 2 lists 34 curated automatable metricswith their attributes including quality dimension, metric name, definition, and whether it is foundational.

**Additional file 3.** Pseudocode. Additional file 3 contains the pseudocode for the tool that implemented quality assessment in this study.

## Data Availability

All data and materials are open resource available on the Web, see specific URIs in https://github.com/sxzhang1201/assess-rdf-resource/blob/master/test%20resources. All intermediate results are either included in the article or in additional files.

## Abbreviations

URI(s): Unique Resource Identifier(s)
RDF: Resource Description Framework
OWL: Web Ontology Language
RDFS: RDF Schema
LDQD: Linked Data Quality Dimension
DQV: Data Quality Vocabulary
DQM: Linked Data Quality Metric
